# Pseudouridine modification in *Caenorhabditis elegans *spliceosomal snRNAs: unique modifications are found in regions involved in snRNA-snRNA interactions

**DOI:** 10.1186/1471-2199-6-20

**Published:** 2005-10-19

**Authors:** Jeffrey R Patton, Richard W Padgett

**Affiliations:** 1 Department of Pathology and Microbiology, University of South Carolina School of Medicine Columbia, SC 29208 USA; 2 Waksman Institute, Department of Molecular Biology and Biochemistry and Cancer Institute of New Jersey, Rutgers University, Piscataway, NJ 08854 USA

## Abstract

**Background:**

Pseudouridine (Ψ) is an abundant modified nucleoside in RNA and a number of studies have shown that the presence of Ψ affects RNA structure and function. The positions of Ψ in spliceosomal small nuclear RNAs (snRNAs) have been determined for a number of species but not for the snRNAs from *Caenorhabditis elegans *(*C. elegans*), a popular experimental model system of development.

**Results:**

As a prelude to determining the function of or requirement for this modification in snRNAs, we have mapped the positions of Ψ in U1, U2, U4, U5, and U6 snRNAs from worms using a specific primer extension method. As with other species, *C. elegans *U2 snRNA has the greatest number of Ψ residues, with nine, located in the 5' half of the U2 snRNA. U5 snRNA has three Ψs, in or near the loop of the large stem-loop that dominates the structure of this RNA. U6 and U1 snRNAs each have one Ψ, and two Ψ residues were found in U4 snRNA.

**Conclusion:**

The total number of Ψs found in the snRNAs of *C. elegans *is significantly higher than the minimal amount found in yeasts but it is lower than that seen in sequenced vertebrate snRNAs. When the actual sites of modification on *C. elegans *snRNAs are compared with other sequenced snRNAs most of the positions correspond to modifications found in other species. However, two of the positions modified on *C. elegans *snRNAs are unique, one at position 28 on U2 snRNA and one at position 62 on U4 snRNA. Both of these modifications are in regions of these snRNAs that interact with U6 snRNA either in the spliceosome or in the U4/U6 small nuclear ribonucleoprotein particle (snRNP) and the presence of Ψ may be involved in strengthening the intermolecular association of the snRNAs.

## Background

Pseudouridine (Ψ) is an abundant modified nucleoside found in RNA, at one time considered the fifth nucleoside of RNA [1]. This modification has been found in many types of RNA and is particularly abundant in small stable RNAs such as transfer RNA (tRNA) and small nuclear RNA (snRNA) [2,3]. Because of its structure, with the carbon at the 5 position of the uracil ring attached to the sugar rather than the nitrogen at the 1 position, Ψ is potentially more versatile in its hydrogen bonding interactions [4]. The presence of Ψ appears to strengthen stems in RNA secondary structures and to stabilize base stacking in loops [5-7]. The splicing of pre-messenger RNAs (pre-mRNAs) in Xenopus oocytes is dependent on the presence of Ψ in U2 snRNA [8,9]. This requirement for Ψ in U2 snRNA was also seen in mammalian splicing extracts [10,11]. In addition, structural studies indicate that Ψ at a particular position in U2 snRNA is necessary to obtain the proper conformation of the pre-mRNA:U2 snRNA interaction during splicing [12-14]. The presence of Ψ at position 34 in human U2 snRNA enhances the formation of a splicing intermediate found when an RNA oligonucleotide containing the lariat branch point of the pre-mRNA is incubated with *in vitro *transcribed U2 and U6 snRNA segments [15].

The formation of Ψ is catalyzed by pseudouridine synthases after transcription and a number of site-specific synthases have been characterized from eukaryotes for the modification of tRNAs [16-22]. It has been shown that the pseudouridylation of several of the residues in metazoan snRNAs require small nucleolar RNA cofactors (snoRNAs) [23-25]. In yeast the formation of Ψ in U2 snRNA is accomplished by a combination of site-specific Ψ synthases [22,26,27] and a synthase that is RNA cofactor dependent [28].

The nucleotides that are modified to Ψ in the spliceosomal snRNAs (U1, U2, U4, U5 and U6 snRNA) have been completely mapped for two species of yeast [26,29] and rat [30]. In addition, the modifications in several of the spliceosomal snRNAs have been sequenced for other species such as fruit fly, frog, humans, and plants (for review see [3]). The positions that are pseudouridylated are, for the most part, conserved and nearly all of the Ψs appear in the 5' half of the snRNAs [3,29,30]. The systematic mapping of the sites of pseudouridylation on these essential splicing cofactors has not been attempted for one of the most popular model systems currently used in biology, *Caenorhabditis elegans *(*C. elegans*). This is especially surprising given the fact that cis-splicing in *C. elegans *exhibits a number of differences from other species. For example, there is a highly conserved 3' acceptor site and a lack of an identified lariat branch point consensus sequence within introns [31,32].

In this report we present the results of the mapping of the sites of Ψ modification on the spliceosomal snRNAs of *C. elegans*, the first step in a larger endeavor to determine the factors required for the formation of Ψ in worms and the function(s) of these modifications in an animal model of development. All of the *C. elegans *spliceosomal snRNAs contain Ψ, which distinguishes these results from those seen with the snRNAs from two yeast species [26,29]. *C. elegans *U2 snRNA is the most highly modified with a total of nine Ψs, all in the first 56 nucleotides of the snRNA. U5 snRNA has three Ψ residues in the highly conserved stem-loop known to be essential for pre-mRNA splicing. *C. elegans *U4 snRNA has two Ψs, with one in a region of the snRNA that participates in an RNA-RNA interaction with U6 snRNA and allows for the formation of the U4/U6 small nuclear ribonucleoprotein particle (snRNP). U1 and U6 snRNAs each have one Ψ identified. When the sites of modification are compared with snRNAs sequenced from other species, two of the positions modified on *C. elegans *snRNAs are unique, one at position 28 on U2 snRNA and one at position 62 on U4 snRNA. These modifications are in regions of each snRNA that are predicted to interact with U6 snRNA to form the U4/U6 snRNP or in the spliceosome during the removal of introns from pre-mRNA [33].

## Results

A primer extension method [34,35] was used to identify the positions of Ψ in the snRNAs. The method involves chemically treating the total RNA from wild type worms with 1-cyclohexyl-3(2-morpholinoethyl) carbodi-imide metho-*p*-toluenesulphonate (CMCT) which covalently reacts with uridines, and other nucleotides, whether modified or not. Two concentrations of CMCT were used in the reactions and the reaction was allowed to proceed such that only a portion of the available uridines is covalently modified. This partial reaction is important since it is essential to be able to extend the primer past the first Ψ encountered in order to identify additional Ψs. The isolated total RNA is then treated with mild base, and as a consequence only the Ψ residues are left covalently modified. This covalent modification of Ψ creates a stop to reverse transcriptase (RT) progression during the specific primer extension phase of the assay. The products are electrophoresed on a 10% polyacrylamide/8.3 M urea gel along with sequencing lanes using the same end-labeled primer. When bands appear in the CMCT+ lanes but not in the CMCT- lanes and those bands are just prior to a uridine in the sequence, then there is a Ψ at that position.

Nearly all of the Ψ residues that have been mapped on any snRNA of any species are found in the 5' half of the RNA [3,30], the exceptions being one residue found near the 3' end of U6 snRNA (rat, mouse, bean) and two residues that are found at positions 131 and 132 on Drosophila U4 snRNA. Therefore this primer extension technique [34,35] can be used to determine the modification state of all but the very 3' 18 nucleotides of the snRNA. To map the positions of Ψ on *C. elegans *snRNAs, primers that hybridized to the extreme 3' end of each snRNA were used initially. Three additional primers were used to map sites of modification on U2 snRNA, and two additional primers were used to map U4, U5, and U6 snRNAs. Only one additional primer was used to map modifications on U1 snRNA.

### U1 snRNA

**Figure 1:**
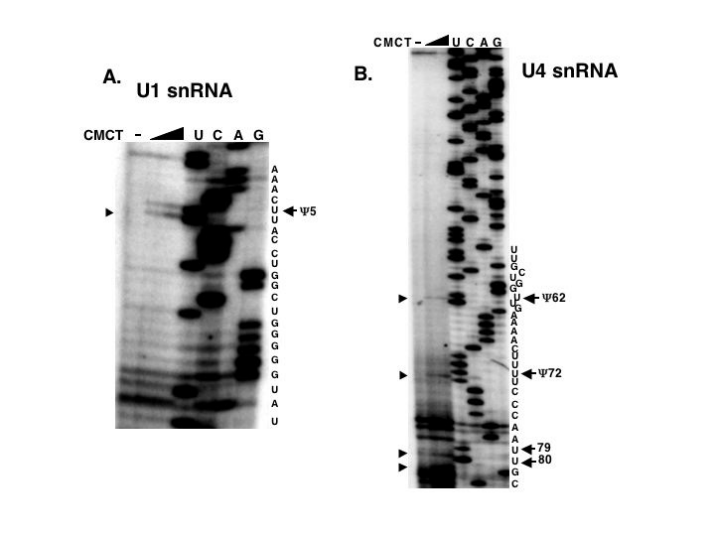
Location of Ψ in *C. elegans *U1 and U4 snRNAs. Total RNA was treated with either 0, 0.041, or 0.167 M CMCT and subsequently treated with mild base (see Materials and Methods). These samples were used as templates in primer extension reactions using ^32^P-end-labeled primers and electrophoresed on denaturing polyacrylamide gels. A portion of the autoradiograph is shown in panel (**A**) and is the result using primer U1RevB. The same primer was also used to generate the sequencing lanes, using *C. elegans *U1 pGEMT plasmid as the template, on the right of the panel. The lanes are labeled to correspond to the RNA sequence. In (**B**) the U4RevB primer was used to determine the positions of Ψ in U4 snRNA using treated RNA samples and a U4 pGEMT plasmid for the generation of the sequence. In this and the subsequent two figures, the arrow to the right of the sequence indicates the position of the Ψ and the arrowhead to the left of the panel indicates a stop to reverse transcriptase that is increased in the CMCT treated lanes. The reverse transcriptase stops just before the modified nucleotide.

With U1 snRNA, only one nucleotide, at the position shown in Figure 1A, was found to be converted into Ψ. This modification is at position 5 of the Thomas et al. [36]* C. elegans *numbering system for U1 snRNA. With the primer extension assay used in these experiments [34], the reverse transcriptase (RT) stops just before the nucleotide that is a Ψ, which is covalently modified by the CMCT. In the case of *C. elegans *U1 snRNA, there is a band before the U at position 5 and not position 6 (Fig. 1A) and therefore position 5 is modified and position 6 is not. The reason for the presence of the double band in the CMCT treated lanes is not known but it has been seen in other substrates by other investigators [37]. Other assays of U1 snRNA using this same primer with a separately isolated total RNA sample and electrophoresed on a different gel also showed this double band. We have noted a double band similar to this previously when Ψ was mapped on *C. elegans *tRNA^Ala ^(AGC) at a position where there was only one uridine [21]. Two primers were used in the primer extension reactions, with one hybridizing to the extreme 3' end of the U1 snRNA (U1RevA) and one closer to the 5' end (U1RevB). The modification shown in Fig. 1A was mapped with this latter primer and U1RevA yielded no additional Ψ residues (data not shown).

### U4 snRNA

U4 snRNAs from other species that have been sequenced have from zero to five Ψs [3,29]. Two Ψs were identified on *C. elegans *U4 snRNA mapped with the U4RevB primer (Fig. 1B). They occur at positions 62 and 72, with stops to RT appearing in the CMCT treated lanes of the gel. Pseudouridines have been found at positions 4, 72, and 79 in all vertebrate U4 snRNAs sequenced, but no other U4 snRNA sequenced has a Ψ at position 62 [3]. The possible modifications of the uridine at positions 79 and 80 of *C. elegans *U4 snRNA, slightly obscured in a region of stops to RT progression even in the CMCT- lane, are marked on the figure. However, the use of additional primers in the assay has not confirmed Ψs at these positions (data not shown). With this specific primer extension method [34] there is a possibility of misidentifying Ψs [38], so since this is a region of considerable secondary structure, the conservative interpretation of the data was that there are no Ψs at these sites. No modification of uridines near the 5' end of *C. elegans *U4 snRNA, such as at position 4 (Fig. 1B), was observed with this or another primer (U4RevC), that is closer to the 5' end of the U4 snRNA (data not shown).

### U2 snRNA

**Figure 2:**
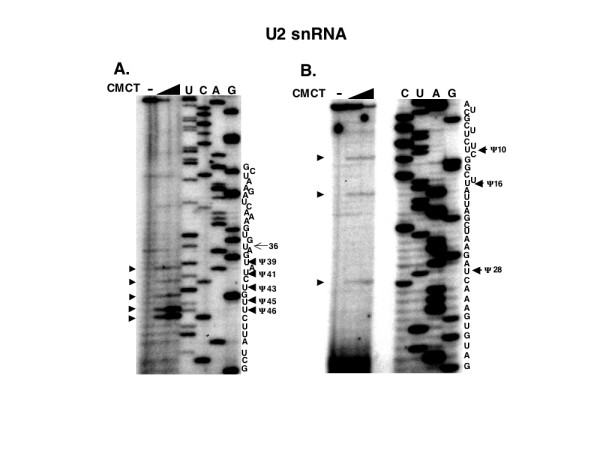
Locations of Ψs in *C. elegans *U2 snRNA. The methods are the same as described in Fig. 1, but the primers used were specific for U2 snRNA and the template for the sequencing reaction was the *C. elegans *U2 pGEMT clone. In panel **A **the primer used was U2RevB and is closer to the 3' end of the U2 snRNA than the primer used in panel **B**, U2RevD. The stick arrow on the right of Panel A shows the position of U36, which is discussed in the text.

As with other U2 snRNAs that have been mapped for modifications [3], *C. elegans *U2 snRNA has the largest number of Ψs in the spliceosomal snRNAs. In a previous report of the characterization of Pseudouridine synthase 1 in *C. elegans *(CePus1p; [21]), we mapped at least five Ψ residues in *C. elegans *U2 snRNA at positions 39, 45, 46, 48, and 56 (*C. elegans *U2 snRNA numbering) using a primer (U2RevC) close to the middle of the snRNA [21]. When another primer (U2RevB) was used instead, two additional modifications, at positions 41 and 43 were seen (Fig. 2A), but the modification at position 48 was not detected. When the assay was repeated using the U2RevC primer used in the earlier report, the modification at position 48 was not detected (data not shown). In Fig. 2, and the other figures, the arrowheads on the left of the panels denote positions just before a U residue where bands occur in both CMCT+ lanes but are missing or greatly diminished in the CMCT- lane. Additional modifications were mapped to positions 10, 16, and 28 (Fig. 2B) of U2 snRNA when a primer closer to the 5' end of the RNA (U2RevD) was used. Three new primers were used to map the Ψ residues on U2 snRNAs in this report and the primer hybridizing to the extreme 3' end of the RNA (U2RevA) did not add any additional pseudouridylated positions to the ones identified with the U2RevB-D primers. This brings the total number of Ψ residues in *C. elegans *U2 snRNA to nine. Surprisingly, no Ψ was detected in *C. elegans *U2 snRNA at the position equivalent to 34 in vertebrates (Fig. 2A, stick arrow on right side of panel; position 36 in *C. elegans *U2 snRNA), even though a Ψ is found at this position in all other U2 snRNAs where the modifications have been determined [3]. This same result was seen with two different primers, there is no increase in band intensity in the CMCT treated lanes, which would indicate a stop to RT and therefore a Ψ (data not shown, see Discussion).

### U5 snRNA

**Figure 3:**
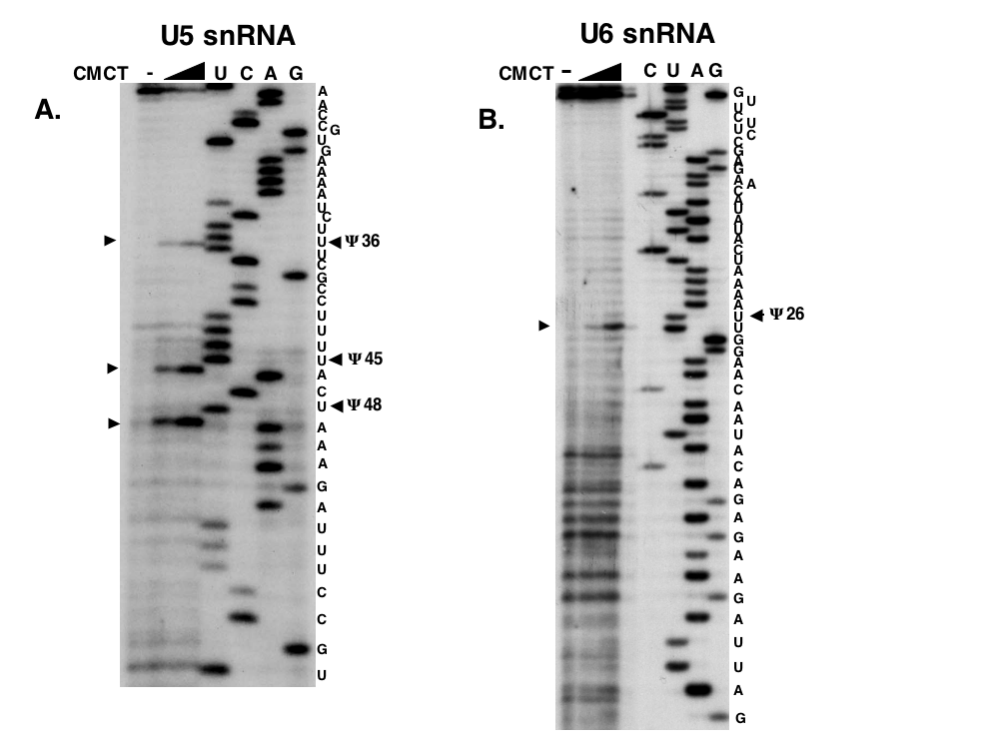
Locations of Ψs in *C. elegans *U5 and U6 snRNAs. The methods are the same as described in Fig. 1, but the primers used were specific for U5 or U6 snRNAs and the template for the sequencing reaction was the *C. elegans *U5 pGEMT or U6 pGEMT clones. Panel **A **shows the results with the U5RevB primer. Panel **B **shows the results of using a primer (U6RevB) that was specific for U6 snRNA and the template for the sequencing reaction was the *C. elegans *U6 pGEMT clone.

**Figure 4:**
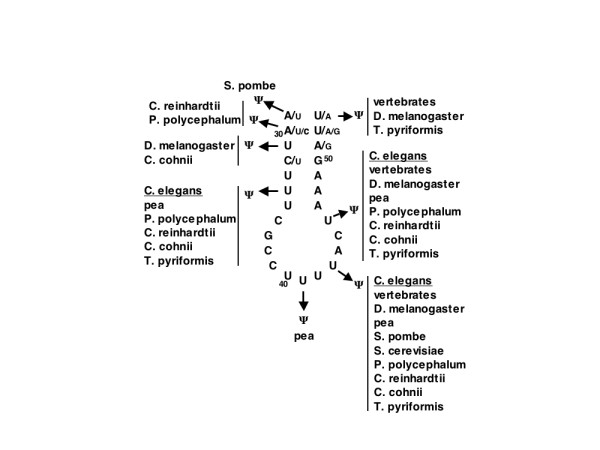
Phylogenetic comparison of the locations of Ψs in sequenced U5 snRNAs. Only the terminal portion of the large stem-loop is shown in the figure. The numbering system and primary sequence corresponds to human U5 snRNA and the positions of the modifications for the other species shown are for the homologous position in the human sequence [3]. At positions 29, 30, 32, 51, 52, and 53 the other nucleotides found at those positions in any of the species are indicated in a smaller font, the rest of the sequence is conserved.

In many of the U5 snRNAs that have been sequenced there are several Ψ residues in the region of the terminal loop of the large stem-loop (see Fig. 4) [3]. Even yeast (*S. pombe*) has two modifications in this region [29], a portion of U5 snRNA that has been shown to be critical for the function of this splicing factor [39,40]. In the U5 snRNAs where Ψs have been found, there is a Ψ at position 43 and most have an additional one at position 46 (see Fig. 4, vertebrate U5 snRNA numbering). *C. elegans *has three Ψ residues in this loop region and the result with the U5RevB primer is shown in Fig. 3A. There are two Ψs at positions 45 and 48 in *C. elegans *U5 snRNA numbering, which correspond to positions 43 and 46 in vertebrates. The third Ψ, shown at position 36 in Fig. 3A, is in the stem at a position that it shares with several other species, including the pea, but not with vertebrates or with either *S. pombe *or *S. cerevisiae *(Fig. 4).

### U6 snRNA

One Ψ was mapped on U6 snRNA from *C. elegans *at position 26 (Fig. 3B), which is equivalent to position 31 in the vertebrate U6 snRNA numbering system. This position is modified in mammals but not in either yeast U6 snRNA mapped [26,29]. A second primer (U6RevC, data not shown) was used to confirm the lack of modification at position 35, which corresponds to a Ψ in mammalian U6 snRNA (position 40). Based on the sequences of other U6 snRNAs [3] there is a possibility that there might be another Ψ residue close to the 3' end of the *C. elegans *U6 snRNA (at position 81). Since even the most 3' primer we used in these assays would hybridize to this position, we were not able to determine if worm U6 snRNA has this modification using this technique.

### CePus1p knockout

In yeast, Pus1p modifies one position on U2 snRNA [26], but when total RNA from CePus1p knockout worms (VC110, see Experimental section) was used as template for the primer extensions with U1RevB, U5RevC, and U6RevC primers there was no difference in the Ψ modification pattern seen with wild type (N2) and VC110 RNA (data not shown). This result was also seen with U2 snRNA from VC110 worms, and was reported previously [21].

## Discussion

In all the snRNAs that have been sequenced and the modifications determined, U2 snRNA has by far the most Ψs. *C. elegans *U2 snRNA has nine, less than the number seen with vertebrates and flowering plants but greater than the number seen with either *S. pombe *or *S. cerevisiae *[3]. The overall modification pattern seen with *C. elegans *U2 snRNA is unique, and there is one position that is modified to Ψ that is not found in the U2 snRNAs from other species (position 28). The other eight modifications were found at sites that have previously been identified as sites of pseudouridylation in U2 snRNA and two (positions 41 and 43 in rat U2 snRNA) have been found in all U2 snRNAs mapped [3]. These modifications have been implicated in the function of the pre-mRNA splicing cofactor [8-11]. It was shown by an *in vitro *reconstitution system for pre-mRNA splicing that the modified U2 snRNA reconstituted splicing but U2 snRNA without Ψs did not [10,11]. In addition, modifications at the 5' end of U2 snRNA were shown to be necessary for the cofactor's activity in the reconstitution of pre-mRNA splicing in *Xenopus *oocytes [8]. Using this same system, Zhao and Yu [9] have recently shown that the presence of Ψ in the region of U2 snRNA (nts. 34–46) that interacts with the pre-mRNA lariat branch point is required for function of the cofactor and for the formation of the active 17S U2 small nuclear ribonucleoprotein particle (snRNP).

The 'branch point interacting region' of U2 snRNA is highly pseudouridylated, with six modifications in vertebrates and *V. faba*, five in *S. pombe *and 3 in *S. cerevisiae *[3]. All of these species have U2 snRNAs that have Ψ at positions 34, 41, and 43. *C. elegans *also has five Ψs in this region, and although there are Ψs at positions equivalent to 41 and 43, we were unable to detect a modification at the position equivalent to 34 (*C. elegans *position 36). The sequence of this region is highly conserved [30], and so even though there is a uridine at position 36 in the U2 snRNA from *C. elegans*, it does not appear to be converted to Ψ. This is a surprising result given the importance attached to the presence of Ψ at this position. It is the nucleotide that is predicted to be directly across from the bulged adenosine in the helix formed by U2 snRNA and pre-mRNA in mammalian and yeast splicing mechanisms [33]. Newby and Greenbaum have shown that the presence of Ψ at this position stabilizes the bulged adenosine conformation in oligomers, which is predicted to be favorable for splicing [12-14]. In addition, Valadkhan and Manley have shown that *in vitro*, a Ψ at position 34 in U2 snRNA enhances the formation of a putative splicing intermediate between U2 and U6 snRNAs [15]. It has been shown recently that when the loss of Pseudouridine synthase 7 (Pus7p), responsible for Ψ35 in *S. cerevisiae *U2 snRNA (the Ψ34 equivalent), is coupled with the mutation of position 40 in U2 snRNA (a U to G substitution or the deletion of the U), a synthetic growth defect is seen. Neither the loss of Pus7p nor either mutation at position 40, showed a phenotype alone. This suggests there is a requirement for the Ψ at position 35 under certain conditions [41].

How is it that worms can do without Ψ at this position? It is possible our assays were incapable of detecting Ψ at this particular position in *C. elegans *U2 snRNA with the primers that we have used. But we think this is unlikely since we identify Ψs on either side of position 36. It is a formal possibility that CMCT does not react with this particular Ψ in worm U2 snRNA and therefore would not result in a stop to RT, but we believe this is also unlikely. Instead an answer might be found in the differences in consensus sequences seen with *C. elegans *introns. There is no identified lariat branch point consensus sequence in *C. elegans *introns and the consensus sequence at the 3' acceptor site is extended and highly conserved [31,32]. Since there is no branch point consensus in worms, there may be no requirement for a Ψ at position 36 in *C. elegans *U2 snRNA. In *C. elegans *U2 snRNP may be more closely associated with the 3' splice site since it has been found that the U2 associated factor (U2AF) is associated with this region of the intron rather than the lariat branch point [32].

**Figure 5:**
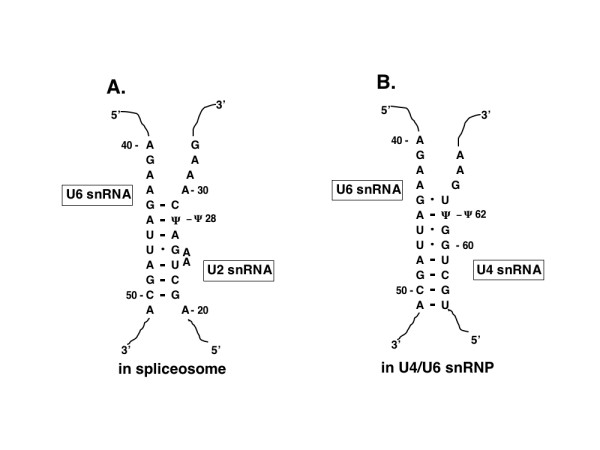
The locations of Ψs in the predicted intermolecular interactions between *C. elegans *U6 snRNA and U2 and U4 snRNAs. Watson-Crick base pairs are denoted with a dash and G:U base pairs denoted with a dot. In **A **the U2-U6 Helix I of the spliceosome [33] is shown. In **B **the interaction between the same portion of U6 snRNA and U4 snRNA in the U4/U6 snRNP is shown.

Although *C. elegans *U2 snRNA does not appear to have a Ψ at position 36 it does have a Ψ at position 28, a modification that occurs in no other sequenced U2 snRNA [3]. This position is significant since it is in a region of *C. elegans *U2 snRNA that is predicted to interact with U6 snRNA in the active spliceosome (see Fig. 5A; [33,42]). The addition of an A-Ψ base pair would add stability to the inter-RNA stem and may favor the adoption of a particular conformation in the U6 or U2 snRNA that would promote catalytic function [5,6,33,43]. It is possible that this modification is critical due to the lack of an extensive lariat branch point interaction between U2 snRNA and the pre-mRNA in *C. elegans *[32].

Of the spliceosomal snRNAs, the modifications on U5 snRNA have been determined from the largest number of species [3]. One position (43, in human U5 snRNA numbering) is converted to Ψ in all of the U5 snRNAs where Ψ has been found (Fig. 4). In addition, many U5 snRNAs, *C. elegans *U5 snRNA included, also have a Ψ at position 46 (Fig. 4). The nucleotide sequence in the loop is highly conserved and is critical for the function of U5 snRNA in the splicing of pre-mRNA [30,39,40]. However, the position of Ψ in the stem is not highly conserved, except for a tendency to be located near the ends of the stem (Fig. 4). In tRNAs, Ψs located at the ends of stems are thought to stabilize base-pairing and therefore strengthen the stem [4]. This could be the reason the actual position is not conserved, only the presence of the Ψ in the stem. Interestingly, *S. cerevisiae *U5 snRNA does not have a Ψ in the stem, only at position 43 in the loop. This yeast has a U5 snRNA that can be folded into a different structure from the other U5 snRNAs [3]. It has an additional stem loop that could have a stabilizing effect on the stem-loop shown in Fig. 4, thereby abrogating the need for the presence of Ψ in the stem in U5 snRNA of *S. cerevisiae*.

*C. elegans *U1 snRNA has two uridines at positions 5 and 6, in this region of U1 snRNA that is essential for the function of the splicing factor, but only the uridine at position 5 is converted to Ψ. Many species, including vertebrates, *Drosophila*, and *S. cerevisiae*, also have two uridines at these positions but both are converted to Ψ. However, algae and *S. pombe *U1 snRNAs have two uridines, and Ψ appears only at the position nearest the 5' end of the RNA [3].

*S. pombe*, and *S. cerevisiae *U4 snRNAs have no detectable Ψs [26,29], whereas vertebrates, *Drosophila*, and *V. faba *(bean), all have several Ψs in their U4 snRNAs [3]. One of the three Ψs found in rat U4 snRNA (at position 4) is in a portion of U4 snRNA that is predicted to interact with U6 snRNA, suggesting the presence of Ψ in this region of U4 snRNA could strengthen the interaction. An equivalent Ψ is not present in *C. elegans *U4 snRNA but the Ψ at position 62, a Ψ that is found in no other sequenced U4 snRNA, is in an area that is predicted to participate in a second intermolecular hybridization with *C. elegans *U6 snRNA (see Fig. 5B). This A-Ψ base pair is in the middle of three G-U base pairs and so would lend greater stability to the stem formed by U4 and U6 snRNAs. This same snRNA-snRNA interaction is predicted to have only one G-U base pair with mammalian U4 and U6 snRNAs [44]. The A residue in *C. elegans *U6 snRNA that the Ψ at position 62 of U4 snRNA pairs with is the same residue that is predicted to base pair with the Ψ 28 of U2 snRNA (Fig. 5A), providing evidence of the need to strengthen each of the snRNA-snRNA interactions that involve this region of U6 snRNA.

## Conclusion

The total number of Ψs found in the snRNAs of *C. elegans *is significantly higher than the minimal amount found in yeasts but it is lower than that seen in sequenced vertebrate snRNAs. When the actual sites of modification on *C. elegans *snRNAs are compared with other sequenced snRNAs most of the positions correspond to modifications found in other species. However, two of the positions modified on *C. elegans *snRNAs are unique, one at position 28 on U2 snRNA and one at position 62 on U4 snRNA. Both of these modifications are in regions of these snRNAs that interact with U6 snRNA either in the spliceosome or in the U4/U6 small nuclear ribonucleoprotein particle (snRNP). These Ψs and may be involved in strengthening the intermolecular association of the snRNAs.

The determination of the Ψ residues in the spliceosomal snRNAs of *C. elegans *will speed the identification of enzymes and cofactors that are involved in the modification of these uridine residues. The location of the modifications will also help in the identification of those cofactors by providing sequences that can be used to predict guide sequences in the snoRNAs. Once we can identify cofactors we will be able to use the powerful genetics of *C. elegans *to mutate the cofactors and affect the formation of Ψ at any position in the snRNAs, especially those that might be critical for the function of the splicing cofactors.

## Methods

All enzymes and the *fmole*^® ^Sequencing Kit were purchased from Promega Corporation (Madison, Wisconsin) except for Taq DNA polymerase, which was purchased from Fisher Scientific. The knockout of the *C. elegans *gene that codes for the homologue of Pseudouridine synthase 1 (Pus1p; strain VC110, W06H3.2 (gk38)) was provided by the *C. elegans *Reverse Genetics Core Facility at the University of British Columbia, which is funded by the Canadian Institute for Health Research, Genome Canada, and Genome BC. This strain was characterized in an earlier publication [21].

The snRNA genes from *C. elegans *were amplified using the polymerase chain reaction (PCR; [45,46]) from genomic DNA using the following primers: for U1 snRNA, 5'AAACTTACCTGGCTGGGGG3' (U1For) and 5'TTCAGGGCCGCGCGCACGC3' (U1RevA); for U2 snRNA, 5'ATCGCTTCTTCGGCTTATTAGC3' (U2For) and 5'ACTGGGCCGAGCCCGGCAGC3' (U2RevA); for U4 snRNA, 5'AGCTTTGCGCTGGGGCGATAACG3' (U4For) and 5'TTCTGCCTCCTAGAGGCGTTC3' (U4RevA); for U5 snRNA, 5'AACTCTGGTTCCTCTGCATT3' (U5For) and 5'TGACCCGCTTTCTCAAGGAG3' (U5RevA); and for U6 snRNA, 5'GTTCTTCCGAGAACATATAC3'(U6For) and 5'AAAAATTTGGAACGCTTCACG3' (U6RevA). The fragments were gel purified, inserted into pGEMT, and sequenced. The sequences of the inserts matched that of the published snRNA genes from *C. elegans *[36].

### Isolation of total RNA from worms and assay for Ψ

Total RNA from N2 worms grown at 20°C on Nematode Growth Media plates was isolated as described [47]. Total RNA (10 μg) was treated with 0, 0.041, or 0.167 M 1-cyclohexyl-3(2-morpholinoethyl) carbodi-imide metho-*p*-toluenesulphonate (CMCT, Aldrich) for 15 minutes at 37°C, precipitated, and treated with sodium bicarbonate as described [34,35]. These treated RNAs (1 μg) were used in primer extension reactions using AMV reverse transcriptase and ^32^P-end-labeled primers as described [35] and the products separated on denaturing (8.3 M urea) 10% polyacrylamide gels (19:1; acrylamide:bis-acrylamide). Sequencing reactions using the same labeled primers were electrophoresed along with the primer extension reactions [35,48]. The reverse primers listed above for the cloning protocol were used first and the additional primers were: for U1 snRNA, 5'GGCCTAACCATGGGGATTCC3' (U1RevB); for U2 snRNA, 5' CATTCGAGTGTATACCGTAG 3' (U2RevB), 5'CCGAGTCTTCCCTAGGTTCC 3' (U2RevC) and 5'CCTTTATTACACTCATTCG3' (U2RevD); for U4 snRNA, 5'GCGTATGCCACCCATGTTTC3' (U4RevB) and 5'TAAACGCACCTCGGCAAAGC3' (U4RevC); for U5 snRNA, 5'GTAATACTCAGTAAATGCTCC3'(U5RevB) and 5'CTCAGTAAATGCTCCTTTGC3' (U5RevC); for U6 snRNA, 5'GCGTGTCATCCTTGCGCAGG3' (U6RevB) and 5'GCGCAGGGGCCATGCTAATC3' (U6RevC). The dried gels were exposed to Kodak XAR film for various lengths of time.

## Authors' contributions

JRP participated in the conception of the study, carried out the mapping of the Ψs in the snRNAs, analyzed the data, wrote the initial draft of and edited the manuscript. RWP participated in the analysis of the data and in the conception of the study, edited the manuscript, provided experience with *C. elegans*, and provided materials for the study.
